# Identifying Children at Risk of High Myopia Using Population Centile Curves of Refraction

**DOI:** 10.1371/journal.pone.0167642

**Published:** 2016-12-28

**Authors:** Yanxian Chen, Jian Zhang, Ian G. Morgan, Mingguang He

**Affiliations:** 1 State Key Laboratory of Ophthalmology, Zhongshan Ophthalmic Center, Sun Yat-sen University, Guangzhou, China; 2 Research School of Biology, Australian National University, Canberra, Australia; 3 Centre for Eye Research Australia, University of Melbourne, Royal Victorian Eye and Ear Hospital, East Melbourne, Victoria; Soochow University Medical College, CHINA

## Abstract

**Purpose:**

To construct reference centile curves of refraction based on population-based data as an age-specific severity scale to evaluate their efficacy as a tool for identifying children at risk of developing high myopia in a longitudinal study.

**Methods:**

Data of 4218 children aged 5–15 years from the Guangzhou Refractive Error Study in Children (RESC) study, and 354 first-born twins from the Guangzhou Twin Eye Study (GTES) with annual visit were included in the analysis. Reference centile curves for refraction were constructed using a quantile regression model based on the cycloplegic refraction data from the RESC. The risk of developing high myopia (spherical equivalent ≤ -6 diopters [D]) was evaluated as a diagnostic test using the twin follow-up data.

**Results:**

The centile curves suggested that the 3^rd^, 5^th^, and 10^th^ percentile decreased from -0.25 D, 0.00 D and 0.25 D in 5 year-olds to -6.00 D, -5.65D and -4.63 D in 15 year-olds in the population-based data from RESC. In the GTES cohort, the 5^th^ centile showed the most effective diagnostic value with a sensitivity of 92.9%, a specificity of 97.9% and a positive predictive value (PPV) of 65.0% in predicting high myopia onset (≤-6.00D) before the age of 15 years. The PPV was highest (87.5%) in 3^rd^ centile but with only 50.0% sensitivity. The Mathew’s correlation coefficient of 5^th^ centile in predicting myopia of -6.0D/-5.0D/-4.0D by age of 15 was 0.77/0.51/0.30 respectively.

**Conclusions:**

Reference centile curves provide an age-specific estimation on a severity scale of refractive error in school-aged children. Children located under lower percentiles at young age were more likely to have high myopia at 15 years or probably in adulthood.

## Introduction

The prevalence of myopia has increased rapidly over the past few decades, especially in East Asia[1–4]. Furthermore, about 10–20% myopes now have high myopia,[5] which carries a much increased risk of complications, such as retinal detachment, glaucoma, cataract and pathological myopia. These disorders cannot be corrected by wearing glasses and often progress to uncorrectable visual impairment and blindness[6,7]. The relationship between severity of myopia and pathological outcomes appears to be highly non-linear, with more severe myopia producing disproportionately increased levels of pathology, emphasizing the importance of limiting the progression of myopia.

In recent years, many groups have reported the efficacy of various treatments in reducing progression of myopia, such as atropine eyedrops[8,9], orthokeratology[10,11], and specialized contact lens[12]. However, all these treatment options may suffer potentially severe side effects [8,13], and thus may raise challenges in clinical practice. It is therefore important that the treatments, particularly the more aggressive treatments with known potential side effects, should target those who are the most at risk of developing high myopia later in life.

Severity of refractive error is generally estimated as a degree of spherical equivalent (SE). In adult, -6 diopters (D) is commonly used as a criterion to define high myopia in adults. However, identifying severe myopia in younger children, as an indicator of risk for subsequent progression to high myopia can be challenging: for example, -2D at 12 years old among Chinese children would not be classified as severe myopia but -2D at 6 years old would be quite severe. The current challenge is how to define proper age-specific cutoffs for the severe myopia to identify children at risk of high myopia and its pathological consequences later in life.

Reference centile curve may provide a good approach to tackle this problem, since it allows estimation on a severity scale based on a population-based age-specific distribution. Such curves have been widely used to describe the distribution of specific characteristics of a population and to assess health status of an individual relative to a reference population. For example, growth reference centiles have been developed since 1891[14] and used to assess obesity, underweight and other growth abnormalities[15–17]. In addition, reference centiles of biometric parameters such as plasma troponin and body temperature are also used to predict certain clinical disorders [18,19]. However, to the best of our knowledge, such curves have not been developed and used in eye care services.

In this paper, we present reference centile charts of the distribution of refraction in children using cross-sectional data from the Guangzhou Refractive Error Study in Children (RESC) study, and validate its accuracy on the estimation of severity and also on the prediction on the onset of high myopia using longitudinal data from the Guangzhou Twin Eye Study (GTES).

## Materials and Methods

### Participants

Cross-sectional data were drawn from 4364 school-age children aged 5–15 years residing in the Liwan district of Guangzhou city as part of the RESC studies. The exclusion criteria for the analysis included children with any tropia, ocular media opacity or history of orthokeratology treatment. Further details of sampling and examination procedures have been described elsewhere[1]. Longitudinal data were drawn from the GTES, which started in 2006 and was followed in annual visits up to 2012. The age of children in GTES ranged from 7 to 15 years at baseline. Details of the sampling and methodology were reported elsewhere [20].

Both the samples were collected in the same city (Guangzhou) in urban South China. The cross-sectional data and longitudinal data were comparably representative of the population in urban area of South China in terms of refractive status and schooling intensity.

### Measures

Refractions was measured using an autorefractor (RESC: ARK-30, Nidek Corp; GTES: KR8800, Topcon Corp, Tokyo, Japan) after cycloplegia. Cycloplegia was induced with 2 drops of 1% cyclopentolate (1% Cyclogyl, Alcon Labs, Fort Wroth, Texas) instilled 5 minutes apart. All examinations for the two studies were carried out by the same research group following the same study protocol. The studies were conducted in accordance with the Tenets of the World Medical Association's declaration of Helsinki. Written informed consents were obtained from all the participants or their parents, and ethical committee approval was got from the Zhongshan University Ethical Review Board and Ethics Committee of Zhongshan Ophthalmic Center

### Statistical analysis

The definition of high myopia was spherical equivalent (SE) -6.00 D or less. SE was calculated as sum of sphere and 1/2 cylinder. Quantile regression, a type of regression model estimating conditional quantile functions, was adopted to construct the reference centile curves on the RESC cross-sectional data.

To validate the accuracy of the age-specific severity estimation in predicting high myopia, baseline centiles and refraction at age 15 years of first-born twins were used as test and outcome variables to conduct diagnostic test. The sensitivity, specificity, positive predictive value (PPV), negative predictive value (NPV) and Mathew’s correlation coefficient (MCC) were calculated. Predictions were made by locating a subject into regions between the quantile curves based on his/her age and SE, to identify if he/she would develop high myopia by the age of 15. All the statistical analysis was performed with Stata (Stata version 12.0, Stata Corp., College Station, TX)[21].

## Results

**Fig 1:**
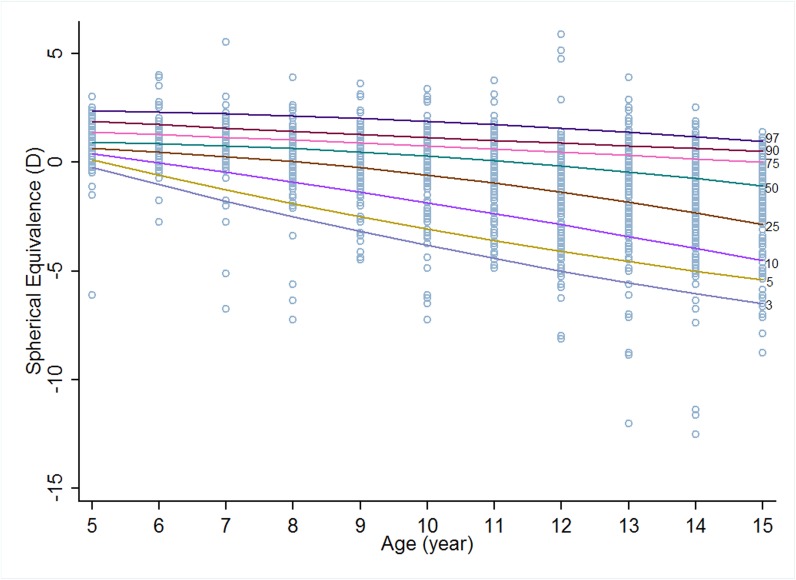
Percentile curves for urban Chinese boys based on cross-sectional data.

**Fig 2:**
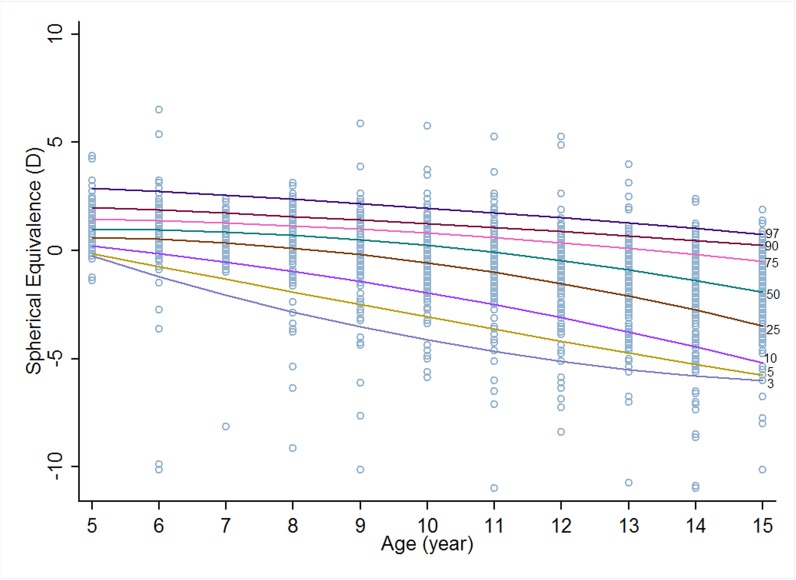
Percentile curves for urban Chinese girls based on cross-sectional data.

Figs 1 and 2 show eight centile curves of refraction reference for boys and girls from RESC at different percentiles (3^rd^, 5^th^, 10^th^, 25^th^, 50^th^, 75^th^, 90^th^, and 97^th^). The 3^rd^ centile of girls tended to be more myopic than that of boys from 6 through 13 years old. For the 50^th^ centile, girls had higher SE from age of 5 to 9 than boys, whereas the trend reversed thereafter. The variation of 97^th^ centile on age was similar to that of the 50^th^ centile (Table 1).

**Table 1:** Refraction (diopter) cutoff of boys compared with that of girls at different age and centile using population-based data of the Guangzhou Refractive Error Study in Children (RESC) study.

Age (year)	Boys			Girls			Difference (girl-boy)		
	3^rd^	50^th^	97^th^	3^rd^	50^th^	97^th^	3^rd^	50^th^	97^th^
**5**	-0.25	0.92	2.38	-0.25	1.00	2.80	0.00	0.08	0.42
**6**	-0.90	0.85	2.26	-1.20	0.96	2.63	-0.30	0.11	0.37
**7**	-1.54	0.75	2.14	-2.06	0.86	2.44	-0.52	0.11	0.30
**8**	-2.17	0.63	2.02	-2.83	0.71	2.25	-0.66	0.08	0.23
**9**	-2.78	0.47	1.89	-3.52	0.50	2.05	-0.74	0.03	0.16
**10**	-3.38	0.29	1.75	-4.12	0.24	1.85	-0.74	-0.05	0.10
**11**	-3.96	0.09	1.61	-4.63	-0.08	1.65	-0.67	-0.17	0.04
**12**	-4.53	-0.14	1.47	-5.05	-0.45	1.44	-0.52	-0.31	-0.03
**13**	-5.08	-0.40	1.32	-5.38	-0.88	1.22	-0.30	-0.48	-0.10
**14**	-5.63	-0.69	1.16	-5.63	-1.36	1.00	0.00	-0.67	-0.16
**15**	-6.15	-1.00	1.00	-5.78	-1.89	0.77	0.37	-0.89	-0.23

**Fig 3:**
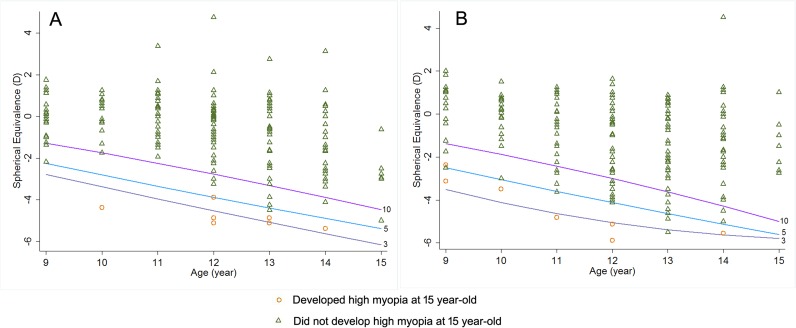
Distribution of age-specific spherical equivalent at baseline and classification of future high myopia onset at 15 years old among first-born twins. A for boys, B for girls. * Centile curves were the ones constructed based on cross-sectional data.

To verify the efficacy of identifying future high myopes using reference centiles, the baseline refraction of 170 boys and 184 girls first-born twins (mean baseline age 11.72 ± 1.60 years and 11.79 ± 1.57 years for boys and girls respectively) was located on the centile curves constructed from the RESC data (Fig 3). Children were divided into non-high myopes and high myopes based on the visit when the children were 15 years old. Those who developed high myopia in the follow-up visits tended to be in the lower centiles (10^th^ and 5^th^).

To estimate the predictive value of different centiles (3^rd^, 5^th^ 10^th^ and 25^th^), we conducted diagnostic tests. Among 354 children, 14 children developed high myopia by the age of 15 years and 15 children became highly myopic after 15 year-old. By using -6.0D by 15 years of age as the outcome of prediction, the 5^th^ and 10^th^ centiles achieved both high sensitivity (92.9% and 100.0%) and specificity (97.9% and 89.1%). PPV values were more than 50.0% when using the 3^rd^ and 5^th^ centiles (87.5% and 65.0%). All the four centiles showed high NPV. MMC was highest using 5^th^ centile as cutoff.

While -6.0D is a commonly used cut-off for the definition of high myopia, the relationship between myopic refraction and pathology starts to rise rapidly from about -4.0D[22]. In addition, many children even after 15 years old will progress further, and become more myopic, and possibly highly myopic, with age. For these two reasons, we also used -5.0 D and -4.0 D before 15 years of age as outcome of interest. To avoid trivial true positive predictions, children with SE ≤ -5.0D or ≤ -4.0D, who were already established high myopia, at baseline visit were excluded.

Using these less stringent cut-offs of -5.0D or -4.0D, the PPV of the 5^th^ centile was higher while the sensitivity was lower. A complete overview of this analysis is shown in Table 2. Among those with -5.0 D and -4.0 D before age of 15 years, only 48.4% and 28.3% of them developed high myopia after the age of 15 years respectively.

**Table 2:** The accuracy of baseline centiles to estimate refractive status at the age of 15 years in first-born twins *.

Baseline centile (%)	True positive (*n*)	False positive (*n*)	False negative (*n*)	True negative (*n*)	Total (n)	Sensitivity(%)	Specificity(%)	Positive predictive value (%)	Negative predictive value (%)	MCC
**Developed ≤-6.00 D at 15 year-old**										
** 3**^**rd**^	7	1	7	339	354	50.0	99.7	87.5	98.0	0.65
** 5**^**th**^	13	7	1	333	354	92.9	97.9	65.0	100.0	0.77
** 10**^**th**^	14	37	0	303	354	100.0	89.1	27.5	100.0	0.49
** 25**^**th**^	14	109	0	231	354	100.0	67.9	11.4	100.0	0.28
**Developed ≤-5.00 D at 15 year-old**										
** 3**^**rd**^	4	0	35	307	346	10.3	100.0	100.0	89.8	0.30
** 5**^**th**^	12	1	27	306	346	30.8	99.7	92.3	91.9	0.51
** 10**^**th**^	29	14	10	293	346	74.4	95.4	67.4	96.7	0.67
** 25**^**th**^	37	78	2	229	346	94.9	74.6	32.2	99.1	0.47
**Developed ≤-4.00 D at 15 year-old**										
** 3**^**rd**^	1	0	55	274	330	1.8	100.0	100.0	83.3	0.12
** 5**^**th**^	6	0	50	274	330	10.7	100.0	100.0	84.6	0.30
** 10**^**th**^	26	2	30	272	330	46.4	99.3	92.9	90.1	0.62
** 25**^**th**^	50	49	6	225	330	89.3	82.1	50.5	97.4	0.58

* Participants with established high myopia (based on the defined cutoffs) at baseline were excluded in this assessment.

MCC: Mathews correlation coefficient

We further evaluated the accuracy in identifying high myopes before age of 15 years at different levels of baseline centiles by sex. For boys, the 5^th^ centile can identify the high myopes before 15 year-old with higher sensitivity, specificity, PPV and MCC. The 5^th^ centile at baseline predicted highly myopic girls with good sensitivity and specificity, but relatively low PPV (50%). A detailed description of the analysis is given in Table 3.

**Table 3:** The accuracy of baseline centiles to identify first-born twins with high myopia (SE ≤-6.0D) at the age of 15 years by sex.

Baseline centile (%)	True positive (*n*)	False positive (*n*)	False negative (*n*)	True negative (*n*)	Total (n)	Sensitivity(%)	Specificity(%)	Positive predictive value (%)	Negative predictive value (%)	MCC
**Boys**										
** 3**^**rd**^	3	0	4	163	170	0.43	1.00	1.00	0.98	0.6
** 5**^**th**^	6	0	1	163	170	0.86	1.00	1.00	0.99	0.9
** 10**^**th**^	7	9	0	154	170	1.00	0.94	0.44	1.00	0.6
** 25**^**th**^	7	42	0	121	170	1.00	0.74	0.14	1.00	0.3
**Girls**										
** 3**^**rd**^	4	1	3	176	184	0.57	0.99	0.80	0.98	0.7
** 5**^**th**^	7	7	0	170	184	1.00	0.96	0.50	1.00	0.7
** 10**^**th**^	7	28	0	149	184	1.00	0.84	0.20	1.00	0.4
** 25**^**th**^	7	67	0	110	184	1.00	0.62	0.09	1.00	0.2

MCC: Mathews correlation coefficient

## Discussion

The current study presents, to the best of our knowledge, the first application of centile curves to quantitative traits in ophthalmic research. This analysis of children’s refractive error based on representative data from urban China aims to identify, while they are still young, the children at increased risk of developing high myopia. In the validation with longitudinal data, the curves showed high efficacy, as measured by diagnostic testing, in predicting high myopia by 15 years. Given the wide use of centile charts in monitoring weight, height and body mass index (BMI) in children, these simple and feasible methods enable scientists to develop a contemporary cross-sectional reference for this specific area of clinical practice based on up-to-date data, and hopefully help identify those who need closer monitoring and more aggressive treatments.

The 3^rd^ through to the 97^th^ centiles were chosen to be consistent with traditional reference centiles charts used in BMI assessment [23]. In this study, however, we found the region below the 5^th^ percentile to be the most accurate to predict high myopia (with prediction sensitivity 92.9% and specificity 97.9%). The baseline SE measures at the 5^th^ percentile provide the best age-specific SE cutoffs for identifying the children with severe refractive error and increased risk of developing high myopia in adulthood. In the present study, 48 children in 1000 were below the 5^th^ percentile, representing a group of children with severe myopia for their age. The young children in this group can be the potential subjects to receive preventive treatments for myopia like atropine, yet those located between the 3^rd^ and 5^th^ centiles may require future follow-up examination to avoid over-treating. Our findings imply that severe myopia with extreme baseline refraction at young ages contributes to the onset of high myopia before 15 years old or probably in adulthood. We provide cut-off values of each age group for school-age children, thereby providing a basis for clinical referral of extreme cases (below the 5^th^ centile).

Based on the performance of reference centiles in the test, we conclude that reference centile curve is a potentially useful indicator for estimating severity of refractive error in specific age and sex groups, with good sensitivity and specificity. However, the fact that the positive predictive value was only 65.0% when 5^th^ centiles were used as cutoffs for all the subjects, suggests that about 35% children classified as positive at baseline will not develop high myopia by age of 15. This lower positive predictive value is clearly associated with the low prevalence of high myopia in the population. One should also note that the prediction efficacy may also subject to the gender of the participants. PPV in girls was 50% but 100% in boys which may indicate progression of refractive error is different between genders, and thus estimation of risk should be ideally performed separately for boys and girls. Further work should be done to improve the PPV, such as including more risk factors in the prediction, i.e., history of myopic parents [24,25], environmental factors such as outdoor activity time [26,27], near work time, [28,29] and optical biometry. Further follow-up to determine progression rates may also improve predictive power[30]. One of our future directions is to incorporate these potential risk factors into our prediction method. While multi-factor predictions models for myopia prediction has been studied elsewhere[31], our aim is to develop multi-factor-adjustable centile curves for prediction. Further work needs to build a more sophisticated model including baseline centile as a major factor, while at the same time also including other variables clinically relevant to myopia.

A limitation of the present study is that the cross-sectional data based on which the centile curves are constructed will be population-, site- and time-specific. The centile curves or distributions constructed from the RESC dataset should not be generalized to use in other populations, except, with due caution, to those with similarly high prevalence of myopia and high myopia such as other cities in mainland China, Hong Kong, Taiwan, South Korea, Japan and Singapore. The range of baseline age in GTES also limited the validation of centile charts in children aged <7 years. In order to construct valid centile curves for use in clinical practice, it is preferable to use the most up-to-date and representative cross-sectional data obtained from their specific population for this effort. For this purpose, we have developed a web-based system (www.myopcentile.org) allowing the investigators to upload their population-based data and generate reference centile curves based on their own cross-sectional data to generate the centile curves valid for their population. The current study is therefore essentially a proof-of-concept for the general methodology, and external testing of our method will be extremely useful.

In conclusion, the reference centile curves provide information on the age- and gender-specific distribution of refraction in urban Chinese children, where the prevalence of myopia and high myopia is high. They may be useful as a scale to estimate the severity of refractive error at a given age in children, and also can be used to predict the children with increased risk of developing high myopia later in life. This tool may provide a basis for identifying the children for whom a more aggressive preventive treatment is appropriate at young age.

## Supporting Information

S1 File Data of RESC study. (XLSX)

S2 File Data of GTES. (XLSX)
